# Molecular Investigations to Improve Fusarium Head Blight Resistance in Wheat: An Update Focusing on Multi-Omics Approaches

**DOI:** 10.3390/plants13162179

**Published:** 2024-08-06

**Authors:** Tiziana M. Sirangelo

**Affiliations:** Division Biotechnologies and Agroindustry, ENEA—Italian National Agency for New Technologies, Energy and Sustainable Economic Development, 00123 Rome, Italy; tiziana.sirangelo@enea.it

**Keywords:** disease resistance, Fusarium head blight, *Fusarium graminearum*, multi-omics, host-pathogen interaction, *Triticum aestivum* L.

## Abstract

Fusarium head blight (FHB) is mainly caused by *Fusarium graminearum* (*Fg*) and is a very widespread disease throughout the world, leading to severe damage to wheat with losses in both grain yield and quality. FHB also leads to mycotoxin contamination in the infected grains, being toxic to humans and animals. In spite of the continuous advancements to elucidate more and more aspects of FHB host resistance, to date, our knowledge about the molecular mechanisms underlying wheat defense response to this pathogen is not comprehensive, most likely due to the complex wheat–*Fg* interaction. Recently, due to climate changes, such as high temperature and heavy rainfall, FHB has become more frequent and severe worldwide, making it even more urgent to completely understand wheat defense mechanisms. In this review, after a brief description of the first wheat immune response to *Fg*, we discuss, for each FHB resistance type, from Type I to Type V resistances, the main molecular mechanisms involved, the major quantitative trait loci (QTLs) and candidate genes found. The focus is on multi-omics research helping discover crucial molecular pathways for each resistance type. Finally, according to the emerging examined studies and results, a wheat response model to *Fg* attack, showing the major interactions in the different FHB resistance types, is proposed. The aim is to establish a useful reference point for the researchers in the field interested to adopt an interdisciplinary omics approach.

## 1. Introduction

*Fusarium* species are very common fungi pathogenic to plants, animals and humans. Molecular approaches and technologies have been very effective in species identification so far, also making it possible to explore the infection effects in different crops caused, for instance, by *Fusarium oxysporum* [1,2,3], *F. verticillioides* [4,5] and *F. graminearum* [6,7].

*F. graminearum* (*Fg*) is the main causal pathogen of Fusarium head blight (FHB), also known as scab, in wheat (*Triticum aestivum* L.) around the world, causing severe damage, with losses in both grain yield and quality [8,9]. The fungus, which is presumably a hemi-biotrophic pathogen, infects wheat heads in the flowering stage and interferes with seed development, destroying cell walls, starch granules, and affecting storage proteins [10]. *Fg* attack leads to contamination of the infected grains with mycotoxins like deoxynivalenol (DON), nivalenol (NIV), or zearalenone (ZEA), classified as type B trichothecenes [11]. DON is the most dangerous, due to its widespread occurrence in high concentrations in plants, and grain contaminated with it may be unsuitable as food resource due to the serious risks to human and animal health [12]. For this reason, governments set upper limits for DON in wheat grain and its products [13].

The resistance to this disease is diverse among different host wheat species, for instance, durum wheat is less resistant than bread wheat. However, no wheat variety possesses immunity against FHB [14]. FHB resistance is a quantitative trait, in fact, wheat morphological and phenological characteristics and the plant growing environment all affect the infection [15]. Wheat height, maturation stage, and flowering time also influence FHB infection [16,17].

FHB resistance in wheat cultivars manifests in several ways and to distinguish among the different resistance types is essential to understand and to explore the underlying defense mechanisms. Schroeder and Christensen [18] first introduced the concept of ‘resistance types’, discriminating between two types of FHB resistance: resistance to early infection (Type I resistance) and resistance to spreading infection (Type II resistance). Subsequently, others types of resistance were defined: Type III to indicate toxin accumulation, Type IV to describe kernel infection, and Type V to correlate infection to yield losses [19]. Type II and III resistance have been extensively explored [13,20], and over 600 loci have been mapped on all 21 wheat chromosomes [21,22]. Among these loci, *Fhb1* [23], *Fhb2* [24] mostly confer Type II resistance, and *Fhb4* [25] and *Fhb5* [26] mainly Type I resistance. The wheat wild relatives also possess other resistance loci, like *Fhb3* [27] from *Leymus racemosus*, *Fhb6* [28] from *Elymus tsukushiensis* and *Fhb7* [29] from *Thinopyrum elongatum*, all conferring Type II resistance, with *Fhb7* also contributing to Type III resistance. Type II and Type III resistance have often interrelated in many studies, and few quantitative trait loci (QTLs) exclusively associated to Type III resistance have been identified [30].

Due to these encouraging achieved results, it is now recognized that breeding FHB-resistant wheat cultivars is one of the most effective means to monitor and combat this disease, and compared with other agronomic practices based, for instance, on chemical control, genetic resistance is the best approach possibly providing durable FHB resistance [8,31]. To date, numerous FHB resistance genes have been identified and explored, and some of them have also been used in wheat breeding practices allowing to improve resistance to *Fg* [8,31]. However, being a trait with strong interactions with the growing environment, research results interpretation is not easy [20]. Additionally, it is difficult to breed resistant wheat cultivars due to the complex genome of this crop and the complicated resistance mechanisms in wheat–*Fg* interaction [32]. 

Omics sciences, such as genomics, proteomics, metabolomics, transcriptomics, and their combined use in an interdisciplinary approach enable meeting these needs, by analyzing diverse dimensions to understand molecular mechanisms underlying several biological pathways, and by identifying correlations between biological processes and metabolic pathways across different omics layers. Multi-omics approaches are proving to be a powerful strategy to elucidate many plants research topics, for instance, the relationships between plant sub-species, the fruit ripening processes, as well the host-pathogen interaction in different plant species [33,34,35,36,37]. In wheat, relevant insights have been obtained by applying these approaches, and advances in the identification of candidate genes, in the elucidation of crucial pathways involved in biotic and abiotic stress responses, as well as in FHB resistance and detoxification molecular mechanisms were achieved [13,32,38,39]. This resulted in the increase in wheat omics resources and devoted databases which can be used by researchers during their studies [39].

Despite these omics advancements we summarize that several FHB resistance molecular mechanisms have not been fully clarified. Furthermore, due to climate changes, such as high temperature and heavy rainfall, FHB has become more frequent and severe worldwide [15] and it is necessary to explore more resistant genes to be used in wheat breeding programs. 

Here, after a brief description of the first wheat immune response to pathogen, we discuss, for each FHB resistance type, the major QTLs and candidate genes, focusing on multi-omics research findings. The state of the art about FHB resistance QTLs and innovative approaches to better understand complex adaptive and functional traits is also discussed. Finally, according to the emerging examined studies and results, a wheat response model to *Fg* attack, in which the major interactions in the different FHB resistance types are underlined, is proposed. The aim is to establish a useful reference point for researchers mainly interested to adopt an interdisciplinary omics approach to study FHB resistance in wheat.

## 2. Wheat Immune Response to *Fg*

The infection, triggering complex biological pathways in wheat crops, starts from the fungal spore binding on the floral tissue and from the subsequent recognition of signaling molecules during the wheat–*Fg* interaction [40]. A large number of pattern recognition receptors (PRRs) is involved in pathogen detection, activating PAMP-triggered immunity (PTI). In wheat, chitin-binding proteins act as PRRs, which either directly or indirectly bind to the pathogen-associated molecular patterns (PAMPs) and induce basal defense response by activating the mitogen-activated protein kinase (MAPK) pathways [40,41]. 

Rapid reactive oxygen species (ROS) production in response to *Fg* attack is critical to establish plant immune responses. In addition to the direct toxic effects on *Fg*, ROS act as cellular signaling molecules to trigger wheat defense responses, such as cell wall strengthening, hormone synthesis, and programmed cell death [42,43].

The salicylic acid (SA) or the jasmonic acid (JA)/ethylene (ET) signaling pathways, which are known to have an antagonistic interaction, are also involved in the activation of plant disease resistance mechanisms [44,45]. SA, which is synthesized via phenylalanine ammonia lyase (PAL) [46], is involved in several defense pathways generally activated by biotrophic pathogens, while JA/ET pathways are usually activated against necrotrophic pathogens [45].

PTI may not constitute a sufficient defense against pathogens that can suppress their responses through effector proteins, of which ~600 effectors secreted by *Fg* have been detected so far [43,47]. Subsequently defense responses called effector-triggered immunity (ETI) are activated, which can also be induced by the SA or JA/ET pathway and are generally able to control specific pathogen attacks [48,49]. In most plants, including wheat, the conserved nucleotide binding site-leucine-rich repeat (NBS-LRR) disease resistance proteins recognize effectors and initiate ETI [50]. Although PTI and ETI involve distinct activation mechanisms, they can converge into similar yet different downstream responses. In fact, complex interactions between PRR-mediated PTI and NBS-LRR-mediated ETI signaling cascades were recently found [51]. This PTI-ETI crosstalk allows an integrative view of the plant immunity system, where ETI does not appears as a separate immune pathway, but rather as an amplification module depending on the PTI machinery, improving its function [51].

Several innovative studies investigated wheat immune response to FHB, including two recent multi-omics investigations. One study [52] confirmed the relevant role of chitin-recognition receptor in FHB defense, and, by using genomic, transcriptomics and proteomics approaches, demonstrated that wheat lines overexpressing the chitin-recognition receptor of *Haynaldia villosa*, a diploid wheat relative, showed enhanced resistance to *Fg*, powdery mildew and yellow rust infection. The second study [53], based on genomics and transcriptomics methods, confirmed the positive role in defense against both *Fg* and *Rhizoctonia cerealis* of wall-associated kinases (WAKs), a class of receptor-like kinases (RLKs) involved in various plant life processes, such as growth, development and pathogen interactions. Particularly, the homeolog of *WAK2* in common wheat, named *TaWAK2A-800*, was identified as a positive regulator of wheat resistance to FHB and its transcript was shown to be significantly upregulated after *Fg* inoculation in the resistant wheat cultivar Sumai3. Furthermore, knocking down this gene compromised wheat FHB resistance, impairing defense pathways induced by chitin [53].

## 3. Type I Resistance

Type I resistance is the resistance to *Fg* penetration at the early infection stage. It is generally measured by spraying a spore suspension on flowering spikes and assessing disease incidence [54]. 

In this phase of the infection, *Fg*-infected wheat spikes showed high expression levels of pathogenesis-related (PR) genes, such as chitinases, glucanases, thaumatin-like proteins genes [44,55,56,57], which are always associated with the induction of SA signaling pathways [58]. 

In *Arabidopsis thaliana* and in wheat, JA-mediated defense pathways were activated as a consequence of *Fg* infection, happening in a later stage of the infection than to the induction of SA [44]. JA it is also known to affect the phenylpropanoid pathway, promoting the accumulation of flavonoids [59]. In Wang et al. [38], combining transcriptomic with metabolomic analysis in Sumai3 and three regionally adapted Canadian wheat cultivars, SA and JA were confirmed to play predominantly positive roles in FHB resistance, while auxin and abscisic acid (ABA) were associated with susceptibility. It was demonstrated that ABA accumulation most likely suppresses FHB tolerance, by inhibiting the phenylalanine (Phe) pathway genes expression, downregulating genes involved in flavonoid and lignin biosynthesis and, consequently, leading to the weakening of the barrier against *Fg* [60]. Phenylalanine, being the precursor of both the phenylpropanoid and flavonoid pathways, was identified as a key FHB resistance metabolite in spikes of wheat near-isogenic lines (NILs) with resistant *Fhb1* allele [59,61]. The results achieved in [60] are consistent with those of a more recent study [62], in which it was demonstrated that ABA acts as a fungal effector and its accumulation leads to increased FHB susceptibility. Similarly, the wheat auxin receptor *TIR1* negatively regulates defense against *Fg*, as showed in an integrated metabolomics-transcriptomics study involving Sumai3, Taimai198, Huaimai33 and JWI cultivars [63]. Conversely, ethylene (ET) appeared to play a dual and ambiguous role during the wheat interaction with *Fg* [38].

*Qfhs.ifa-5A* from a Sumai3 cultivar has been among the most studied QTLs and was mainly associated to the Type I resistance, also contributing to Type II resistance [64]. 

Kugler et al. [50] investigated a gene co-expression network activated in response to *Fg* using RNA-Seq data from wheat NILs carrying *Qfhs.ifa-5A* (Type I) and *Qfhs.ndsu-3BS*, also known as *Fhb1* (Type II). Transcripts associated with either QTL within the network were identified and the predominant role in the *Fg* response of some gene families, including glucanases, NBS-LRR, WRKY transcription factors and uridine diphosphate (UDP)-glycosyltransferases, was underlined. 

A transcriptomic characterization of these two major *Fg* resistance QTLs [65] allowed also to identify candidate genes and, according to Kugler et al. [50], an UDP-glycosyltransferase gene was detected, exhibiting a positive difference in response to *Fg* in lines harboring both QTLs compared to lines carrying only the *Qfhs.ifa-5A* resistance allele. Furthermore, in NILs coming from a cross of the susceptible spring wheat cv. Remus and the FHB-resistant line CM-82036, hosting both QTLs, ~15 transcripts showed a significantly different response for *Fhb1* and ~350 for *Qfhs.ifa-5A*. 

These two QTLs, known to be very relevant for wheat FHB resistance response, were also investigated in bread wheat. In a study [66], an integrated transcriptomics and metabolomics approach was applied in wheat NILs to dissect the molecular response to *Fg* and the DON toxin. Results underlined how the infection affects the glutamate metabolism in bread wheat lines hosting *Qfhs.ifa-5A*. A receptor-like protein kinase, a protein kinase, and an E3 ubiquitin-protein ligase were also detected among candidate genes only expressed in *Fhb1*. Furthermore, tricarboxylic acid cycle (TCA) genes such as aconitases, citrate synthase, and succinate dehydrogenases along with malic enzymes showed greater expression level. 

Recently, to characterize the genetic basis of FHB resistance, phenotypic and gene expression data coming from ~95 European winter wheat genotypes, representing a wide variety in FHB resistance, were analyzed [67]. Several resistant genes appeared to be shared among different genotypes, suggesting that the basal/initial defense response mechanisms are largely independent from the line resistance degree. The wheat lines from Sumai3 showed a higher expression of genes associated with cell wall production and of genes involved in terpene metabolism. Furthermore, the *Qfhs.ifa-5A* gene expression analysis detected a NST1-like protein gene, generally involved in stress response, as a candidate gene for Type I resistance. 

In the same year, stably expressed Type I (*Qfhi.nau-2D*) and Type II resistance (*Qfhs.nau-2A*) QTLs were identified from two Yangmai 158 (Y158) recombinant inbred line (RIL) populations, where Y158 is a resistant elite wheat cultivar mainly cultivated in China [68] (Table 1). 

## 4. Type II Resistance

Type II resistance is generally defined as the resistance to the spread of infection within the plant tissues, which, based on a large number of observations, are not pervaded before 36 h after *Fg* inoculation [54]. Type I and Type II resistance cannot be well distinguished by spray inoculation, where disease severity is used as a measure of overall FHB resistance. Type II resistance is then detected injecting a spore suspension into individual florets (point inoculation) and calculating the percentage of visually infected spikelets [54].

This resistance type is considered more stable compared to the other types; for this reason, it has been largely investigated and the related research results have been applied in many breeding programs [69]. 

*Fhb1* especially provides durable and stable Type II resistance to FHB and it is the most important and widely studied QTL [23]. Many wheat varieties grown throughout the world host the *Fhb1* QTL, and this helps to reduce the severity of the infection in many cases [15].

In *Fhb1*, a pore-forming toxin-like (PFT) gene, encoding a chimeric lectin protein, where lectins are often associated with biotic and abiotic responses in plants, was first identified [70]. Recently, Chen et al. [71] demonstrated that in wheat the suppression of the expression of the *TaJRLL1* and *Ta-JA1/TaJRL53* genes, encoding jacalin-related lectins, another type of chimeric lectin protein, reduced FHB resistance, while overexpressing *TaJRL53* enhanced it, most likely through the binding to oligosaccharides characterizing the infection process.

Furthermore, genomic and gene expression investigations identified in *Fhb1* a *TaHRC/His* gene, encoding the histidine-rich calcium-binding protein, located adjacent to PFT [72,73,74]. This gene is a susceptible gene, and a deletion in its codon region silences it, resulting in FHB resistance [72,73]. However, how *HRC/His* affects FHB resistance has not been fully elucidated. Only recently, a study [75] led to the identification in wheat of two HRC variants which drive liquid–liquid phase separation (LLPS) within a nuclear proteinaceous complex with opposite effects, causing FHB resistance or susceptibility. It seems that in wheat susceptible HRC possesses stronger LLPS ability than resistant HRC. However, other investigations are needed to clarify the role of HRC in wheat defense response.

Other multi-omics studies were carried out to investigate the resistance mechanisms conferred by *Fhb1*. For instance, combining metabolomic and proteomic approaches, QTL in NILs coming from the Nyubai wheat genotype were investigated [76]. The comparison of the metabolomic and proteomic profiles revealed that the phenylpropanoid pathway plays an important role in FHB resistance and confirmed that cell wall thickening and flavonoids are also responsible for this resistance [76].

Subsequently, the role of *Fhb1* during the *Fg* infection in NILs derived from Sumai3 and Stoa cultivars, hosting resistant and susceptible alleles, respectively, was examined, and DON accumulation as well as transcriptomic response were analyzed [77]. In addition to the identification of a set of FHB and DON-responsive genes in *Fhb1*, results showed that rachis is a crucial location for Type II resistance.

The relevant role of lignin, a primary component of the cell wall, in wheat FHB defense was confirmed in several recent studies [78,79]. In Soni et al. [78], the combination of metabolomics and genomics analysis identified laccase as one of the key genes in *Fhb1* of wheat NILs coming from Sumai 3*5 and Thatcher cross. In Gao et al. [79], the expression of genes involved in plant defense was investigated in XN979, a FHB-resistant wheat cultivar. Results showed that the FHB resistance of this cultivar is mainly based on of two different defense pathways. The first pathway is constituted by genes involved in lignin and JA biosynthesis being highly expressed during *Fg* infection, while the second one is related to genes involved in biotic stress response.

The role played in *Fg* infection by lignin and caffeoyl-coenzyme A O-methyltransferase (CCoAOMT), which participates in lignin biosynthesis, was also recently investigated in wheat [80]. Here, ~20 CCoAOMT genes were characterized through a genome-wide analysis method. Furthermore, their expression values and co-expression network were analyzed using a RNA-Seq approach. By qRT-PCR validation, potential candidates defense genes were identified. Finally, on the basis of resequencing data, the genetic diversity of these genes was studied in bread wheat and its relatives. By sequencing and phylogenetic analysis, another recent study about *Fhb1* [81] investigated the role of the NAC transcription factor, which was often reported to regulate the lignin biosynthesis [82,83] and, by a transcriptomics analysis revealed a higher expression of *TaNAC032* in resistant NILs. About NAC genes and their role in wheat FHB resistance, a proteomics and gene expression analysis also showed that the gene *TaNACL-D1*, encoding a Triticeae-specific protein C-terminal region, interacts with an orphan protein and enhances resistance to FHB [84].

Multi-omics studies were also carried out about *FHb2* QTL. For instance, an integrated metabolomics-transcriptomics approach revealed FHB candidate resistance genes in wheat [85]. The metabolomics analysis revealed abundant phenylpropanoid, lignin, glycerophospholipid and flavonoid compounds, whose pathways are more active in wheat-resistant RILs than in susceptible ones, and the transcriptomics analysis underlined the overexpression of several genes, such as receptor kinases, transcription factors and DON detoxification genes. Furthermore, the *Fhb2* resistance was associated with high rachis resistance based on cell wall enforcement and DON detoxification genes [85]. 

Another QTL was largely investigated for FHB resistance in wheat, the *2DL QTL*, and numerous FHB-resistant wheat lines from a Chinese germplasm collection have been shown to possess this QTL [86]. In a recent study, by using RNA-Seq analysis, differentially expressed genes (DEGs) were detected from wheat NILs carrying this QTL [87]. Taking into account these studies and by using QTL and RNA-Seq analyses, ~25 DEGs located on chromosome arm 2DL were selected for further characterization [88]. Results showed that only two of them are located near the 2DL QTL: a gene annotated as WD40 repeat family protein, and a gene encoding a SAM-dependent methyltransferase (S-adenosyl methionine-dependent methyltransferase) which is known to play a key role in the phenylpropanoid, flavonoid and other secondary metabolic pathways [86]. Located in this QTL, using metabolomics, gene expression and QTL analyses, another study identified several resistant FHB genes in wheat, including *TaACT* genes, encoding agmatine coumaroyl transferase, where feruloylagmatine appears to inhibit spore germination but not mycelium growth of *Fg* [89]. Furthermore, it was found that *TaACT* reinforces the secondary cell wall by accumulating hydroxycinnamic acid amides (HCAAs), of which some metabolites, such as coumaroylagmatine and coumaroylputrescine, characterized by high expression values during *Fg* infection, were identified in *Fhb1*-resistant wheat genotypes by a multi-omics analysis, combining metabolomics and quantitative real-time PCR methods [90]. A WRKY like transcription factor, the *TaWRKY70* gene, which increases the accumulation of HCAAs, was also identified in *2DL* QTL, and was validated with a gene silencing-based approach [90].

*FHb7*, another QTL mainly associated to Type II resistance and showing a stable resistance against *Fg*, was first transferred from *Thinopyrum ponticum* to common wheat [29] and cloned in recent years, by using a reference assembly generated for *Th. elongatum*, which hosts biotic resistance genes and is a great resource for wheat breeding [91]. However, the lack of a *Thinopyrum* reference genome hinders gene cloning and markers identification, reducing the number of *Fhb7* research programs. *Fhb7* was also found to encode a glutathione S-transferase (GST) which can act against trichothecenes produced by *Fg* [91]. Remarkably, wheat lines hosting *Fhb7* showed increased resistance to FHB without influencing wheat growth and its yield [91], making *Fhb7* a promising QTL for wheat resistance breeding (Table 2).

## 5. Type III Resistance

Low mycotoxin accumulation is referred to as Type III resistance [92], which some researchers considered as a component of Type II resistance as it contributes to the infection spread decrease [93].

Several studies summarize the molecular networks involved in the mycotoxins production during *Fg* infection and the methods for their detoxification, providing useful inputs for crop improvement programs [94,95]. Many of them reported that toxin accumulation is inversely correlated to FHB resistance. For instance, a recent comparative transcriptomic analysis, involving wheat cultivars with different FHB resistance and characterized by specific defense mechanisms, underlined that toxin accumulation is inversely proportional to their FHB resistance level [96]. Therefore, reducing toxin accumulation, especially DON, is not only appropriate to safeguard human health, but also a crucial aim of FHB resistance research activities. 

Other investigations reported QTL associated with FHB severity and at the same time with DON accumulation in wheat [20,97]. Conversely, QTLs exclusively associated with DON accumulation have been reported in a few studies. Among them, one study [30], using a RIL population coming from a cross between spring wheat lines ‘NASMA’ and ‘IAS20*5/H567.71’, demonstrated the presence of two QTLs on chromosomes 3BL and 3DL, mainly resulting into DON reduction, and with minor or no influence on FHB resistance, suggesting that DON accumulation resistance and FHB resistance could involve a different set of genes. Furthermore, in the next years, by using two wheat crossings, Anahuac 75 × BR 18-Terena and BR 18-Terena × BRS 179, another QTL analysis was carried out. ~15 QTLs associated with FHB symptoms were identified, and among them two QTLs mainly related to DON accumulation were detected on chromosome 4B and 6B [98].

Many other studies were focused on proteins involved in DON detoxification and their identification. 

The potential role of the ribosomal protein L3 (RPL3) gene family in DON resistance of wheat has been discussed for some time now and it was found that one of the genes encoding this protein, the *TaRPL3-A3* gene, maps the *Qfhs.ifa-5A* QTL, typically associated to Type I resistance [99]. 

An ATP-binding cassette (ABC) transporter gene was found to contribute to DON tolerance in wheat [100], and was also characterized and located by PCR-based mapping and by sequence analyses on the short arm of wheat chromosome 3B, while gene expression analysis showed its activation as an early host response to DON and to the FHB defense hormone JA. 

Cytochrome P450 enzymes that can be involved in several kinds of oxidation–reduction reactions and pathways, including that relating to lignin biosynthesis, were also investigated and a study revealed that *TaCYP72A* gene is able to catabolize the DON mycotoxin in wheat and can be activated under DON treatment and *Fg* infection [101]. 

Uridine diphosphate (UDP)-glycosyltransferases (UGTs) have been widely reported to be able to detoxify DON. For instance, by a genomics–transcriptomics analysis, the *TaUGT5 gene*, located on chromosome 2B, was characterized and reported to reduce DON accumulation in wheat [102]. Similarly, by a multi-omics approach based on genomics, transcriptomics and metabolomics analysis, *TaUGT6* was characterized and its positive role in reducing DON content was confirmed [103]. Furthermore, transgenic durum wheat plants expressing the barley *HvUGT13248* gene and bread wheat plants expressing the same transgene in flower tissues were generated and by combining genomics, transcriptomics and metabolomics approaches, it was demonstrated that under *Fg* infection, FHB symptoms have decreased in both transgenic wheat [104]. In addition, results showed that DON detoxification may also limit *Fusarium* crown rot (FCR) caused by *F. culmorum*, and thus, it can be considered a trait of interest for wheat research programs concerning FHB as well as FCR resistance. In the same year, it was demonstrated that the *Brachypodium distachyon* UGT *Bradi5gUGT03300* can confer FHB resistance in wheat, and its introduction in the variety Apogee showed an increased FHB resistance and a strong reduction in DON content in infected spikes [105]. The study, using metabolomics and transcriptomics analysis, also confirmed that wheat Type II and Type III resistance to FHB are, in this case, strongly correlated.

About GST proteins, in addition to Wang et al. [91] study, another recent research underlined the relevant role of GSTs in FHB infection in wheat and, more specifically, by using transcriptomics and proteomics analyses, demonstrated that *Fg* inoculation results in the overexpression of several GST genes, including *TaGSTU120*, possibly playing a role in the systemic response to the infection, and *TaGSTF26* which could have an important role in the successful defense [106] (Table 3).

## 6. Type IV Resistance

Type IV resistance is about the kernel infection whose visual symptom is quantified by estimating the percentage of Fusarium-damaged kernels (FDK) in a sample [107]. It was often confused with Type II resistance, also concerning possible *Fusarium* kernel damages [108,109]. Type IV is poorly understood by researchers, for instance, it is not clear why some wheat cultivars have a poorer FDK than what is expected assessing their Type I and Type II resistance levels [110]. This could mean that there are QTLs for FDK that are not associated with Type I or Type II resistance, suggesting that these genes are specifically associated to these types of resistance, as reported in a research work [111]. Conversely, many mapping studies reported that QTLs for Type I or Type II resistance also affect FDK or DON content in wheat [108,112]. 

A recent study tried to find relationships between the different types of FHB resistance in wheat [113]. Here, the reaction of winter wheat lines to FHB were evaluated after *F. culmorum* inoculation, another pathogen causing this disease. *Fusarium* biomass was examined using real-time PCR, while trichothecenes B accumulation was analyzed by gas chromatography techniques. Furthermore, a significant correlation was found between FHB infection symptoms and FDK, which was reported to be mainly related to NIV content.

While the correlation between Type IV and Types I and II is not completely clear, the one with Type III resistance appears more defined. In fact, many researchers investigating the correlation between FDK and DON, considered FDK as a consequence of the presence of mycelium within the grain, mainly leading to DON production [114,115]. Recently, Gaire et al. [115] used FDK as a trait to perform genomic selection analysis, finding that it is the most important secondary trait to predict DON. Furthermore, in Berraies et al. [116] from an adapted durum wheat population, four DON accumulation resistance QTLs were identified on chromosomes 1A, 5A and 7A, two FDK resistance QTLs were identified on chromosomes 5 and 7A, and candidate genes which could be involved in FDK and DON resistance were detected within these QTLs regions (Table 4).

## 7. Type V Resistance

Type V resistance correlates FHB infection to yield losses. Miller et al. [92] and Mesterházy et al. [19] were the first researchers to investigate it, but subsequently this type of resistance was poorly studied probably because of the significant overlaps with the other types. Recently, Gong et al. [117] proposed to merge Type V into Type IV resistance since Type IV resistance necessarily influences the grain yield.

Interestingly, a recent study [118] demonstrated that some winter wheat cultivars can show both high yield and high resistance, suggesting that breeding FHB-resistant wheat cultivars does not necessarily result in yield losses.

## 8. Quantitative Traits Loci Associated with *Fg* Resistance: Other Relevant Analysis Approaches

From the state of the art and the studies discussed in the previous section, it is clear that breeding for FHB resistance by using QTL/genes is one of the effective approaches to control this disease and to hamper toxins contamination in wheat. Furthermore, fine mapping has led to the identification of many QTLs associated with FHB from several resistant wheat cultivars, such as Sumai 3 (*Fhb1*, *Fhb2*, *Rht1*, *Qfha.ifa-5Ac*, *Qfha.ifa.5AS*, *Qfhs.ndsu-3BS*), Wangshuibai (*Fhb1*, *Fhb2*, *Fhb4 Fhb5*, *Qfhi.nau-4B*), and Yangmai 158 (*QFhb-5A*) [40], of which *Fhb1*, from Chinese germplasm, has stable characteristics in different wheat development environments [16]. However, most FHB resistance QTLs do not have this characteristic and are population specific [119]. 

To identify stable QTLs controlling FHB resistance, the QTL meta-analysis approach, which has been applied for several years, making it possible to integrate QTLs studies in a consensus wheat reference genome, can help focus on given QTL regions and detect candidate genes [120]. 

On the other hand, advancements in the sequencing of wheat genome, such as the ‘Svevo’ durum wheat genome [121], enabled identifying consensus genomic regions, as well as to investigate relationships among candidate genes within QTLs. Recently, significant studies based on these findings and using combined omics approaches have been carried out [21,22,122]. In Venske et al. [21], a meta-analysis carried out starting from a large bread wheat QTL collection has generated a smaller set of FHB-resistant meta-QTLs, and enabled finding markers more closely linked to these regions. Furthermore, the integration of these analysis with genomic and transcriptomic data enabled identifying and confirm FHB-resistant candidate genes for breeding programs, like those encoding a glycosiltransferase and a Cytochrome P450. In Soriano et al. [122], comprehensive QTL meta-analysis and transcriptomics investigations were carried out on ~40 traits of durum wheat, including quality, stress-related and FHB resistance traits. Interestingly, results showed that some traits are always associated, such as FHB, GPC (grain protein content) and YPC (Yellow pigment content), and this could help identify and characterize genes with a pleiotropic effect on yield and quality traits. In Zheng et al. [22], meta-QTLs were located on the Chinese Spring reference genome and high-confidence QTLs were selected. Locus-specific single-nucleotide polymorphisms (SNP) and genes responsive to FHB were then identified by combining transcriptomics and proteomics data. Results indicated a reduction in glycerides during early *Fg* infection and suggested that FHB significantly modified the nitrogen metabolism pathway in wheat. 

Overall, the integration of meta-QTLs analysis with omics enabled defining an innovative molecular approach for improving wheat resistance to FHB.

Another approach to increase FHB resistance consists in pyramiding resistance genes. For instance, in a recent study [123], three QTLs, *Fhb1*, *Fhb4*, and *Fhb5*, were introduced into modern Chinese wheat lines with different environmental characteristics by using a marker-assisted approach. Results showed that the FHB disease degree is decreased compared to that of each wheat line. Other positive effects of the pyramiding approach were observed in other recent studies, involving *Fhb1*, *Fhb2* and *Fhb5* pyramiding in winter wheat [124] and QTL from *T. aestivum*, *T. dicoccum* and *T. dicoccoides* in durum wheat [125].

Overall, it is crucial to identify additional FHB resistance sources and loci for gene pyramiding, as well as to analyze large collections of widespread wheat varieties and elite breeding lines for FHB resistance.

Genome-wide association studies (GWAS) and genomic selection (GS) [126,127] are powerful approaches which meet these needs, and enable identifying promising QTL contributing to the different types of FHB resistance, due to the abundant genetic variation analyzed across wheat broad-spectrum accessions [128,129,130]. Therefore, these two approaches are increasingly evolving into a great tool in many breeding programs in the last decade, and the FHB resistance in wheat is probably one of the most studied research topics [131].

## 9. Conclusions

In recent years, omics technologies have been largely applied on FHB-resistant and susceptible wheat genotypes. Fine mapping of wheat genomic regions has led to the identification of many FHB resistance QTLs, transcriptomic analyses helped detect genes involved in disease resistance and toxins detoxification, and metabolomics and proteomics also contributed to elucidate wheat resistance mechanisms. The combination of these technologies in a multi-omics approach made it possible a deeper and more interdisciplinary knowledge of the molecular mechanisms underlying FHB resistance in wheat, as demonstrated by the significant results examined in this work. 

**Figure 1:**
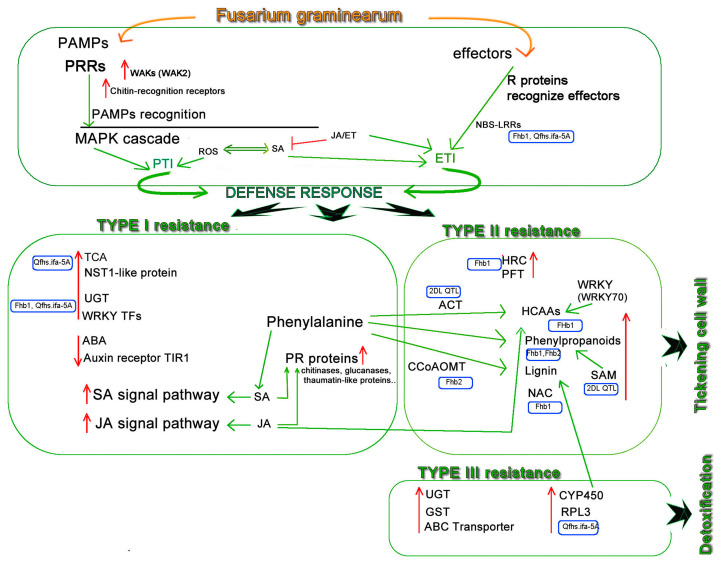
A model of wheat immune and defense responses to *Fg* infection. The immune response and the three types of FHB resistance (Type I, II, III) are grouped (in green rounded rectangles). Arrows (in green) indicate defense response mechanisms associated with different pathways. Rounded rectangles (in blue) are QTLs. The vertical upward arrows represent a positive correlation with resistance and the vertical downward arrows a negative correlation. Open blocks (in red) indicate a negative regulation. PAMPs, pathogen-associated molecular patterns; PRRs, pattern recognition receptors; WAKs, wall-associated kinases; PTI, PAMP-triggered immunity; ETI, effector-triggered immunity; MAPK, mitogen-activated protein kinase; SA, salicylic acid; JA, jasmonic acid; ROS, reactive oxygen species; NBS-LRR, nucleotide binding site-leucine-rich repeat; PR, pathogenesis-related proteins; TCA, tricarboxylic acid cycle; NST1-like protein; UGT, UDP-glucuronosyltransferase; WRKY TFs; ABA, Abscisic acid; HCAA, Hydroxycinnamic acid amide; auxin receptor TIR1; ACT, agmatine coumaroyl transferase; CCoAOMT, caffeoyl-coenzyme A O-methyltransferase; HRC, histidine-rich calcium-binding protein; PFT, pore-forming toxin-like; HCAAs, hydroxycinnamic acid amides; SAM, S-adenosyl methionine; ABC transporter, GST, glutathione S-transferase; CYP450, cytochromes P450; RPL3, ribosomal protein L3.

Figure 1 shows a model of immune and defense responses in wheat–*Fg* interaction, obtained taking into account the studies discussed in this review. It summarizes the initial plant pathogen response generally shared among plants and previously discussed in Section 2. Then, the subsequent defense response is detailed, and the involved protein/genes family grouped according to the major *Fg* resistance types (Type I, Type II and Type III resistance), examined in Section 3, Section 4 and Section 5. Below, the explanation of the mechanisms illustrated in this model is reported.

In the initial defense response, plants rely on PRRs to recognize the pathogen, a mechanism activating, in turn, PTI. Two important PRRs, chitin recognition receptors and WAKs (the *WAK2* gene) are involved in the *Fg* defense response in wheat. ROS production and MAPK also induce PTI. Effector proteins are the other type of initiators. ETI, induced by the interactions of R proteins (e.g., NBS-LRR proteins, which were detected in wheat genotypes carrying *Qfhs. ifa-5A* and *Fhb1*) can start the second line of host-induced defense responses. SA and JA/ET signaling pathways are involved in PTI and ETI activation, stimulating downstream transcription factors and wheat defense responses. 

In Type I resistance, SA and JA were confirmed to play predominantly positive roles in FHB resistance, whereas ET play an ambiguous role during the interaction with *Fg*. For this reason, ET is not reported in this part of the schema. SA is synthetized via the phenylalanine pathway and JA is involved in the activation of the phenylpropanoid pathway. Auxin (the auxin receptor *TIR1*) and ABA were associated with susceptibility. At this stage, *Fg* infected wheat spike expresses elevated levels of PR proteins, like chitinases, glucanases, thaumatin-like proteins, whose expression is always associated with the induction of SA signaling pathways. Other proteins, such as UGT (*Fhb1*, *Qfhs. ifa-5A*), TCA (*Qfhs. ifa-5A*), NST1-like proteins (*Qfhs. ifa-5A*), WRKY Tf (*Fhb1*, *Qfhs. ifa-5A*), also play a positive role in FHB resistance.

In Type II resistance, it is underlined how the phenylpropanoid pathway enhances resistance by thickening cell wall. Phenylalanine, which is the precursor of phenylpropanoid pathways, was identified as a FHB resistance metabolite in wheat. The expression of CCoAOMT (reported in *Fhb2*), involved in lignin biosynthesis, is induced in an early stage of infection, and ACT (*2DL* QTL), associated to a wheat FHB resistance candidate gene, reinforces the secondary cell wall by depositing HCAAs. HCAAs (*Fhb1*), one of the end products of the phenylpropanoid pathway, is then showed, together with the *WRKY70* gene (*2DL* QTL) which contributes to increase their accumulation. Metabolites belonging to the phenylpropanoids pathway were detected in *Fhb1* and *Fhb2* QTLs. SAM-dependent methyltransferase, which is known to play a key role in the phenylpropanoids pathway, is associated with *2DL* QTL. Lignin, which aggregates at the site of *Fg* infection to constitute a barrier and thus to hamper the FHB spreading, is reported in *Fhb1* and *Fhb2*. Its biosynthesis is also regulated by the NAC transcription factor, which is also able to act against FHB infection (*Fhb1*). In the *Fhb1* region, a *PFT* gene, encoding a chimeric lectin protein, was first identified and, subsequently, a *TaHRC/His* gene, encoding a histidine-rich calcium-binding protein, was found, adjacent to *PFT*.

In Type III resistance, gene families involved in *Fg* mycotoxin detoxification, such as UGT, GST, ABC transporters, CYP450, and RPL3, are shown, where CYP450s may be involved in the lignin biosynthesis pathway. It is possible to observe that some genes, such as UGT, may be involved in different FHB resistance types. 

The discussed studies and the schema of Figure 1 could be a reference point for researchers interested in investigating FHB molecular mechanisms in wheat. We hope that this overview can be increasingly enriched due to the continuous improvement of multi-omics research in the field, the spread of the genetic resources, the innovative sequencing technologies, as well as the recent cloning and transgenic techniques.

## Figures and Tables

**Table 1:** Some emergent multi-omics studies about Type I resistance to FHB in wheat.

Type I Resistance	Omics Sciences	Wheat Cultivars/Species	QTL Analysis	Short Description	References
	Genomics, transcriptomics	CM-82036 and Remus	*Fhb1*, *Qfhs.ifa-5A*	The predominant role of glucanases, NBS-LRR, WRKY transcription factors and UDP-glycosyltransferases in pathogen response was underlined	[50]
	Genomics, transcriptomics	CM-82036 and Remus	*Fhb1*, *Qfhs.ifa-5A*	After inoculation with *Fg* spores, LTP and UDP genes were detected	[65]
	Genomics, transcriptomics, metabolomics	Bread wheat	*Fhb1*, *Qfhs.ifa-5A*	Results showed glutamate metabolism changes in lines hosting *Qfhs.ifa-5A*. TCA genes showed greater expression levels, playing important roles in the early stage of *Fg* infection.	[66]
	Transcriptomics, metabolomics	Sumai3 and three regionally adapted Canadian cultivars		Investigation of the role of plant hormones during the interaction of wheat with *Fg*	[38]
	Genomics, transcriptomics	European winter wheat genotypes (including Sumai3)	*Fhb1*, *Qfhs.ifa-5A*	The Sumai3-derivative lines showed higher expression of genes associated with cell wall and terpene metabolism. In *Qfhs.ifa-5A*, a gene encoding a stress response NST1-like protein was identified	[67]
	Transcriptomics, metabolomics	Sumai3, Taimai198, Huaimai33 and JWI		Wheat auxin receptor *TIR1* negatively regulates defense against *Fg*	[63]

**Table 2:** Some emergent multi-omics studies about Type II resistance to FHB in wheat.

Type II Resistance	Omics Sciences	Wheat Cultivars/Species	QTL Analysis	Short Description	References
	Genomics, transcriptomics	Chinese Spring	*Fhb1*	A *TaHRC/His* gene, encoding histidine-rich calcium-binding protein, was identified in *Fhb1*	[72,73,74]
	Metabolomics, proteomics	Nyubai genotype	*Fhb1*	Hydroxycinnamic acid amides and flavonoids, played an important role in FHB resistance	[76]
	Genomics, transcriptomics, metabolomics	Sumai 3, Stoa	*Fhb1*	Results showed that the rachis is a crucial location for Type II resistance	[77]
	Genomics, transcriptomics	Sumai3*5, Thatcher	*FHb1*	The role of laccase gene for FHB resistance is underlined	[78]
	Genomics, transcriptomics	Chinese Spring, Sumai3, Thatcher	*Fhb1*	The role of NAC transcription factor, regulating the biosynthesis of lignin, is relevant in resistance to FHB infestation	[81]
	Transcriptomics proteomics	CM82036, Fielder		The gene *TaNACL-D1* interacts with an orphan protein and enhances resistance to FHB	[84]
	Genomics, transcriptomics		*Fhb2*	The lignin and CCoAOMT role in FHB resistance was investigated	[80]
	Transcriptomics, metabolomics	BW-278, AC Foremost	*Fhb2*	Phenylpropanoids, lignin, flavonoids, mycotoxin detoxification proteins are involved in FHB response	[85]
	Genomics, transcriptomics	HC374, CDC Alsask	*2DL*	~25 DEG located on chromosome arm 2DL were selected. SAM-dependent methyltransferase genes were identified	[88]
	Genomics, transcriptomics, metabolomics	BW301, HC374	*2DL*	Several genes conferring resistance to FHB, including *TaACT* encoding agmatine coumaroyl transferase were identified	[89]
	Transcriptomics, metabolomics	BW301, HC374	*2DL*	HCAAs were identified as resistance metabolites in rachis. *TaWRKY70* transcription factor regulates the biosynthetic of these genes	[90]
	Genomics, transcriptomics	*Th. elongatum*	*FHb7*	*Fhb7* was transferred from *Thinopyrum* and was cloned	[91]

**Table 3:** Some emergent multi-omics studies about Type III resistance to FHB in wheat.

Type III Resistance	Omics Sciences	Wheat Cultivars/Species	QTL Analysis	Short Description	References
	Genomics, transcriptomics	CM82036, Remus		The *TaABCC3.1* gene, associated with DON resistance in wheat, was characterized	[100]
	Genomics, transcriptomics	CM82036, Remus		The *TaCYP72A* gene was found to be activated by DON treatment and *Fg* infection	[101]
	Genomics, transcriptomics	Sumai3, Ning7840, Apogee73S2, Shannong22, Jimai22, Apogee, Liangxing66, Kenong199, Jiyin1, Chinese Spring		The *TaUGT5* gene was characterized and reported to be effective in reducing DON content	[102]
	Genomics, transcriptomics, metabolomics	Sumai 3, Annong 8455, Fielder		The *TaUGT6* gene was characterized and its positive role in reducing DON content in wheat was confirmed	[103]
	Genomics, transcriptomics, metabolomics	Transgenic durum and bread wheat plants		FHB symptoms were reduced in two transgenic wheat plants, obtained introducing the barley *HvUGT13248*	[104]
	Transcriptomics, metabolomics	Apogee		The *Brachypodium distachyon* UGT *Bradi5gUGT03300* confers FHB resistance in wheat	[105]
	Transcriptomics, proteomics	Suma3 and a crossing inbred population of GK Mini Manó/Nobeokabozu		Systemic changes in many elements of the antioxidant/detoxification defense system are detected, and the positive role of GSTs in FHB resistance was underlined	[106]

**Table 4:** The most recent omics studies about FHB Type IV resistance in wheat.

Type IV Resistance	Omics Sciences	Wheat Cultivars/Species	Short Description	References
	Transcriptomics, metabolomics	winter wheat	Fusarium biomass was analyzed. Significant correlation was found between head infection symptoms and FDK	[113]
	Genomics	soft red winter wheat (SRWW)	Results showed that FDK is the most important secondary trait to predict DON	[115]
	Genomics	durum wheat	DON accumulation resistance QTLs and FDK resistance QTLs were identified	[116]

## Data Availability

Not applicable.

## Abbreviations

**Abbreviation**	**Definition**
ABA	Abscisic Acid
ABC transporters	ATP-Binding Cassette Transporters
ACT	Agmatine Coumaroyl Transferase
CS	Callose Synthase
CCoAOMT	Caffeoyl-Coenzyme A O-Methyltransferase
CAD	Cinnamyl Alcohol Dehydrogenase
CYP450	Cytochromes P450
DEGs	Differentially Expressed Genes
DON	Deoxynivalenol
ET	Ethylene
ETI	Effector-Triggered Immunity
GS	Genomic Selection
GST	Glutathione S-Transferase
GWAS	Genome-Wide Association Studies
HCAA	Hydroxycinnamic Acid Amide
HRC	Histidine-Rich Calcium-binding protein
JA	Jasmonic Acid
LEA	Late Embryogenesis Abundant proteins
LRR	Leucine-Rich Repeat
LTP	Lipid Transfer Protein
MAPK	Mitogen-Activated Protein Kinase
MTAs	Marker-Trait Associations
NBS	Nucleotide Binding Site
NILs	Near-Isogenic Lines
NIV	Nivalenol
PAL	Phenylalanine Ammonia Lyase
PAMPs	Pathogen-Associated Molecular Patterns
PCR	Polymerase Chain Reaction
Phe	Phenylalanine
PFT	Pore-Forming Toxin-like
PR	Pathogenesis-Related protein
PRRs	Pattern Recognition Receptors
PTI	PAMP-Triggered Immunity
QTL	Quantitative Trait Loci
RIL	Recombinant Inbred Line
RLKs	Receptor-Like Kinases
ROS	Reactive Oxygen Species
RPL3	Ribosomal Protein L3
SA	Salicylic Acid
SAM	S-Adenosyl Methionine
SNP	Single-Nucleotide Polymorphism
TCA	Tricarboxylic Acid Cycle
UGT	UDP-Glucuronosyltransferase
WAKs	Wall-Associated Kinases
